# Treating Prostate Cancer by Antibody–Drug Conjugates

**DOI:** 10.3390/ijms22041551

**Published:** 2021-02-04

**Authors:** Matteo Rosellini, Matteo Santoni, Veronica Mollica, Alessandro Rizzo, Alessia Cimadamore, Marina Scarpelli, Nadia Storti, Nicola Battelli, Rodolfo Montironi, Francesco Massari

**Affiliations:** 1 Division of Oncology, IRCCS Azienda Ospedaliero-Universitaria di Bologna, 40138 Bologna, Italy; matteorosellini92@gmail.com (M.R.); veronica.mollica7@gmail.com (V.M.); rizzo.alessandro179@gmail.com (A.R.); 2 Oncology Unit, Macerata Hospital, 62100 Macerata, Italy; nicola.battelli@sanita.marche.it; 3 Section of Pathological Anatomy, School of Medicine, United Hospitals, Polytechnic University of the Marche Region, 60126 Ancona, Italy; a.cimadamore@staff.univpm.it (A.C.); m.scarpelli@staff.univpm.it (M.S.); r.montironi@staff.univpm.it (R.M.); 4 Direzione Sanitaria Azienda Sanitaria Unica Regionale, 60122 Ancona, Italy; nadia.storti@sanita.marche.it

**Keywords:** prostate cancer, antibody drug conjugates, target therapy, PSMA, STEAP1, TROP2, CD46, B7-H3

## Abstract

Prostate cancer is the most frequent malignancy in the worldwide male population; it is also one of the most common among all the leading cancer-related death causes. In the last two decades, the therapeutic scenario of metastatic castration-resistant prostate cancer has been enriched by the use of chemotherapy and androgen receptor signaling inhibitors (ARSI) and, more recently, by immunotherapy and poly(ADP–ribose) polymerase (PARP) inhibitors. At the same time, several trials have shown the survival benefits related to the administration of novel ARSIs among patients with non-castration-resistant metastatic disease along with nonmetastatic castration-resistant cancer too. Consequently, the therapeutic course of this malignancy has been radically expanded, ensuring survival benefits never seen before. Among the more recently emerging agents, the so-called “antibody–drug conjugates” (ADCs) are noteworthy because of their clinical practice changing outcomes obtained in the management of other malignancies (including breast cancer). The ADCs are novel compounds consisting of cytotoxic agents (also known as the payload) linked to specific antibodies able to recognize antigens expressed over cancer cells’ surfaces. As for prostate cancer, researchers are focusing on STEAP1, TROP2, PSMA, CD46 and B7-H3 as optimal antigens which may be targeted by ADCs. In this paper, we review the pivotal trials that have currently changed the therapeutic approach to prostate cancer, both in the nonmetastatic castration-resistant and metastatic settings. Therefore, we focus on recently published and ongoing trials designed to investigate the clinical activity of ADCs against prostate malignancy, characterizing these agents. Lastly, we briefly discuss some ADCs-related issues with corresponding strategies to overwhelm them, along with future perspectives for these promising novel compounds.

## 1. Introduction

Prostate cancer is the most frequent malignancy in the worldwide male population as well as one of the most common among all the leading cancer-related death causes [1]. It has been estimated that the rapid decreasing trend of prostate cancer incidence started in 2010 will continue until 2019 and then slow down and reach a plateau after 2050, with several differences among ethnicities [2]. Although several men receive an early-stage disease diagnosis and run an indolent course, many cases are characterized by locally advanced or metastatic disease, at the time of diagnosis. Considering the worst course of the advanced disease, a hard challenge for clinicians has been to find a therapeutic approach for these patients in order to improve their quoad vitam prognosis as much as possible. 

Focusing on metastatic disease, the therapeutic landscape has been evolving over the last decades for both hormone-sensitive (mHSPC) and castration-resistant prostate cancer (mCRPC), leading to genomics-oriented and other novel treatments [3,4]. Furthermore, great progress has also been made in treating the so-called nonmetastatic castration-resistant disease (nmCRPC) from the year 2018 onwards, as described below.

Several studies highlighted the key role played by the androgen-receptor (AR) axis in prostate cancer pathogenesis and subsequent progression, explaining the rationale of the use of androgen-deprivation therapy (ADT) as upfront treatment in metastatic prostate malignancy [5,6]. Moreover, the administration of secondary hormonal manipulation, including –steroidal anti-androgens such as bicalutamide, is used to strengthen ADT based on gonadotropin releasing hormone (GnRH) agonist [7]. 

In the last two decades, the therapeutic scenario of mCRPC has been enriched by the growing body of evidences on the use of chemotherapy and androgen receptor signaling inhibitors (ARSI) in this setting. More recently, immunotherapy and poly(ADP–ribose) polymerase (PARP) inhibitors have been shown to be effective in specific subpopulations of patients with prostate cancer. Among the emerging agents, the so-called “antibody–drug conjugates” (ADCs) are novel compounds consisting of cytotoxic agents (known as “payloads”) linked to specific antibodies able to recognize antigens expressed over cancer cells’ surface. This approach ensures minimal exposure of healthy tissue to cytotoxic agents, expanding the therapeutic window of ADCs [8]. In this review, we illustrate the current landscape of prostate cancer and the potential role of ADC in the future therapeutic armamentarium for this disease. We performed a research on PubMed/Medline, Cochrane library and Scopus using the keywords, “prostate cancer systemic treatment“, or, “nonmetastatic castration-resistant prostate cancer treatment”, or, “metastatic hormone-sensitive treatment”, or, “metastatic castration-resistant prostate cancer treatment”, or, “antibody–drug conjugates”, or, “STEAP-1” OR “TROP2” OR “PSMA” OR “CD46”, or, “B7-H3”. We selected pivotal registration studies. We also selected the most relevant and pertinent studies considering quality of the studies in terms of their applicability, how they were conducted, statistical analysis, number of patients enrolled, outcomes. For ongoing clinical trials, we searched in the clinicaltrials.gov database for recruiting and active, not recruiting trials, using the following keywords: “metastatic prostate cancer”, or, “metastatic castration-resistant prostate cancer”, and, “antibody drugs conjugates”, or, “STEAP-1”, or, “TROP2”, or, “PSMA, or, “CD46”, or, “B7-H3”. We restricted our research to phase one, two or three trials focused on the metastatic castration-resistant setting. 

## 2. Current Therapeutic Landscape of nmCRPC

Particular attention should be paid to recent therapeutic developments in nmCRPC. As mentioned before, ADT is the backbone treatment of advanced disease, but patients may also take advantage of it in case of PSA rising after local treatment in a nonmetastatic setting. If disease relapses despite castrate levels of testosterone induced by “salvage” ADT effect, a castration-resistant state can be recognized even if there is no evidence of secondary lesions. In more detail, the current definition of nmCRPC consists of progression during ADT with no detectable disease in local lymph nodes, bone or visceral organs by computed tomography (CT) or magnetic resonance imaging (MRI). Although there is still no accepted ubiquitary definition of progression on ADT, the Prostate Cancer World Group 3 (PCWG3) consensus has unveiled that the evidence of a 25% increase from the nadir PSA value (in light of a starting PSA level of 1 ng/mL and with a minimum rise of 2 ng/mL), may indicate biochemical relapse, considering castrate levels of testosterone (<50 ng/mL) [9,10]. Certainly, patients who received a similar diagnosis are characterized by meaningful risk of metastatic dissemination and cancer-related mortality, pointing out the need for decreasing metastatic progression risk by ensuring new treatment strategies [11]. Until 2018, the most common therapeutic approach for nmCRPC was observation based on biochemical evaluation (PSA doubling-time) as well as serial imaging, with ongoing ADT. In the last two years, the therapeutic scenario in nonmetastatic castration-refractory setting has been renewed by three phase III trials, which showed the key-role of new-generation ARSIs such as enzalutamide, apalutamide and darolutamide (Table 1). Owing to survival benefits obtained among men with metastatic CRPC before or beyond taxane chemotherapy as mentioned below, enzalutamide was one of the first –steroidal anti-androgens assessed in the nonmetastatic context. The PROSPER trial was a 2018 phase III study, designed in order to compare enzalutamide in combination with ADT to ADT plus placebo in patients with nmCRPC [12]. The preliminary data showed how the administration of enzalutamide 160 mg/die plus ADT was correlated with prolonged metastasis-free survival (MFS) as well as favorable safety profile, ensuring US Food and Drug Administration (FDA) and European Medicines Agency (EMA) approval for nmCRPC treatment in 2018. More recently, Stenberg et al. have reported at ASCO-GU 2020 results from the final analysis of overall survival (OS), highlighting a significant 27% lower risk of death among men in experimental arm [13]. Regarding apalutamide, the use of this AR axis inhibitor for nmCRPC patients was investigated in relevant phase III SPARTAN trial, after having achieved promising data among men with nonmetastatic castration-refractory disease considered characterized by “high-risk of progression” (PSA ≥ 8 ng/mL or PSA doubling-time ≤ 10 months) in a previous phase II trial [14]. Apalutamide 240 mg/die added to ongoing ADT resulted to be associated with significantly improved MFS and time to symptomatic progression in patients with high-risk nmCRPC at the first interim analysis, revealing an OS gain (25% reduction in risk of death if compared with placebo) with mature data of the 2019 final analysis [15,16]. Due to above-mentioned survival benefits, this agent got approval by FDA and EMA in this context. The third study that has allowed the further extension of this therapeutic landscape is the randomized phase III ARAMIS trial, which focuses on the novel anti-androgen darolutamide. As shown by 2019 interim analysis data first and then confirmed by final analysis results presented at ASCO-GU 2020, the addition of darolutamide 300 mg BID to ADT has ensured a gain in terms of prolonged MFS as well as significant OS advantage among patients with a high-risk nonmetastatic disease [17,18]. Moreover, if darolutamide is compared to the above-described ARSIs, it seems to be related with a better safety profile and fewer adverse events [19]. Of note, fatigue, hypertension but also mental impairment were the most common registered adverse events among patients treated with novel ARSIs, if compared with men in control arms (Table 1). Although the safety profile for all these drugs is manageable, the above-described effects appeared to be more noticeable with enzalutamide and apalutamide. In July 2019, FDA has granted approval for the administration of darolutamide in high-risk nmCRPC patients. Nonetheless, this agent has not received EMA approbation in the same setting yet.

## 3. Current Therapeutic Landscape of mCRPC

Until the beginning of the 2000s, the first available cytotoxic chemotherapy to treat advanced ADT-refractory disease was mitoxantrone. This type II topoisomerase inhibitor was approved in 1996 by the US Food and Drug Administration (FDA) because of its improved palliation in symptomatic cases of mCRPC, even if there was no difference in terms of OS between men treated or not treated with mitoxantrone [20].

The meaningful change in this therapeutic field occurred in 2004, in which two phase III studies showed how the administration of taxane chemotherapy leads to benefits in terms of prolonged OS among patients with mCRPC (Table 2) [21]. First of all, in the TAX 327 randomized trial, treatment with docetaxel every three weeks, or weekly, plus prednisone was compared with mitoxantrone plus prednisone [22]. On the other side, the comparison between docetaxel plus estramustine and mitoxantrone plus prednisone was investigated in the Southwest Oncology Group (SWOG) 99–16 randomized trial [23]. In both studies, the use of docetaxel administered every three weeks reached survival benefit, with a median OS gain of 1.9 to 2.4 months: owing to these data, docetaxel-based regimen has been approved as the new standard of care in the castration-resistant setting, especially among symptomatic men.

More recently, with the phase III E3805/CHARTEED study, the use of docetaxel has been expanded to the hormone-sensitive setting [24]. Sweeney et al. proved that six cycles of docetaxel (instead of ten cycles as used in castration-resistant disease) at the beginning of ADT for metastatic HSPC brought more significant survival improvement than that with ADT alone. Of note, this benefit was more evident as well as long-term maintained among men with high disease burden (“high-volume disease” according to CHARTEED criteria), i.e., with visceral metastases or ≥4 bone lesions with ≥1 beyond axial skeleton. Therefore, docetaxel has been approved by the FDA as well as the European Medicines Agency (EMA) to treat patients with a “high volume” castration-sensitive disease.

A further breakthrough in the use of taxane chemotherapy against advanced disease occurred in 2010, when de Bono et al. published the results of the phase III TROPIC study. These data demonstrated the survival benefit among men with progressive post-docetaxel mCRPC treated with cabazitaxel [25], leading FDA and EMA to approve the administration of cabazitaxel in the docetaxel-refractory disease setting. Moreover, the subsequent CARD trial revealed the improvement in terms of several clinical outcomes using cabazitaxel in mCRPC patients who had been previously treated with docetaxel and an androgen-signaling-targeted agent [26].

Beyond chemotherapy, it is well known that the AR axis still plays an essential role in prostate cancer progression, despite the ensured castration state [36]. Since 2012, ARSI have been holding a more and more relevant role in advanced disease therapy, in order to achieve maximal androgen blockade by antagonizing AR function or by inhibiting neoplastic androgen synthesis. Abiraterone acetate is a selective inhibitor of CYP17A1, and it has proven able to gain OS and PFS improvement in patients previously treated with docetaxel in the phase III COU-AA-301 trial, compared with prednisone alone [27]. Furthermore, the phase III COU-AA-302 trial showed similar benefits also among chemotherapy-naive patients with mCRPC [28,29]. Moreover, as highlighted by the 2017 LATITUDE trial, abiraterone acetate is a favorable approach also among men with “high-risk” mHSPC (i.e., Gleason score ≥8, ≥3 bone lesions or visceral metastasis) [30]. This result was enforced by a recent analysis of the abiraterone acetate arm data of the MRC phase III STAMPEDE trial, too [37]. Enzalutamide is a targeted AR inhibitor which has unveiled its clinical activity in both docetaxel-refractory and untreated metastatic CRPC, according to AFFIRM and PREVAIL studies respectively, encouraging its approval in these settings [31,32]. Like abiraterone acetate, also enzalutamide has recently been tested in the hormone-sensitive metastatic context in order to define possible improved clinical outcomes among these patients, in both ARCHES and ENZAMET trials [38]. While phase III ARCHES study has first compared the addition of enzalutamide to ongoing ADT to ADT plus placebo [33], ENZAMET study has investigated enzalutamide plus ADT versus first-generation –steroidal anti-androgen (such as bicalutamide) plus ADT, focusing on different primary end-points (rPFS and OS respectively) [34]. The preliminary results of both these trials hint that enzalutamide has a significant clinical activity in this setting too. As a consequence of the data of these two studies, in August 2019 this new anti-androgen has obtained priority review for supplemental use in the metastatic castration-sensitive disease by US FDA [39].

Regarding adverse events (AEs), abiraterone acetate and enzalutamide are characterized by a safety profile that has less impact on patients’ quality of life than taxane chemotherapy regimens. Hematopoietic and gastrointestinal toxicities of any grade (but especially high grade) were more pronounced among men treated with docetaxel or cabazitaxel, as shown in the corresponding pivotal trials. On the other side, ARSIs appeared to be correlated with cardiocirculatory toxicity as well as other AEs including fatigue, electrolyte alterations or seizures, allowing their preferential use among patients unfit for chemotherapy (Table 2) [5,21].

Currently, the above-mentioned ARSIs along with docetaxel, cabazitaxel and the alpha emitter radium-223 dichloride, correspond to the best treatment against mCRPC in Western countries. Nonetheless, the pursuit of better durable disease control has brought physicians to broaden their therapeutic horizons. The focus has lately shifted to tumor genomic profiling, in order to discover novel biomarkers to predict response, and to expand this wide therapeutic scenario. PARP inhibition has been investigated as a therapeutic strategy against tumors with alterations in DNA damage repair (DDR) genes, including BRCA1/2, ATM, CHEK2, as well as homologous recombination repair (HRR) genes. Large-scale next-generation sequencing (NGS) data detected these genetic defects within a conspicuous part of mCRPC patients [4,40]. The potential benefit of PARP inhibitors (such as olaparib) in prostate cancer has been recently confirmed by phase III PROfound trial (Table 2). These results point out an increasing PFS related to olaparib administration among men with mCRPC already treated with an ARSI and harboring HRR/DRR genes alterations, if compared with enzalutamide or abiraterone acetate [35]. Of note, olaparib clinical activity was highlighted both before and after taxane chemotherapy, as well as among men without BRCA1/2 gene mutations, unveiling the need for further gene-level confirmation studies.

Moreover, nowadays it is known that several DDR-genes defects may lead to a high mutational burden, with the subsequent expression of malignant neoantigens that increases tumor-infiltrating lymphocytes. This drove the experts to investigate the role of immune checkpoint inhibitors (ICIs) against mCRPC. High microsatellite instability (MSI) expression may be a consequence of mismatch-repair (MMR) genes mutations, and may characterize about 3% of mCRPC men [41]. The detection of MSI can predict a high response rate to antiprogrammed cell death protein-1 (PD-1) pembrolizumab in many solid tumors, including prostate cancer [42]. Owing to these data, the US FDA has recommended pembrolizumab for all solid malignancies characterized by MMR mutations and/or high MSI expression, basing on the first ever tissue-agnostic approval [4,43].

Several trials are ongoing nowadays, designed for studying multi-drugs treatment approach, such as PARP inhibitors plus ARSI (i.e., talazoparib plus enzalutamide), ICIs plus PARP inhibitors (i.e., pembrolizumab plus olaparib), etc.

The therapeutic landscape against mCRPC has been widely expanded due to all the above-mentioned agents without any doubt, but the long-term survival benefit of these is still restricted, hinting the need of modern and more effective pharmaceuticals.

## 4. Treating Prostate Cancer by ADC

**Figure 1:**
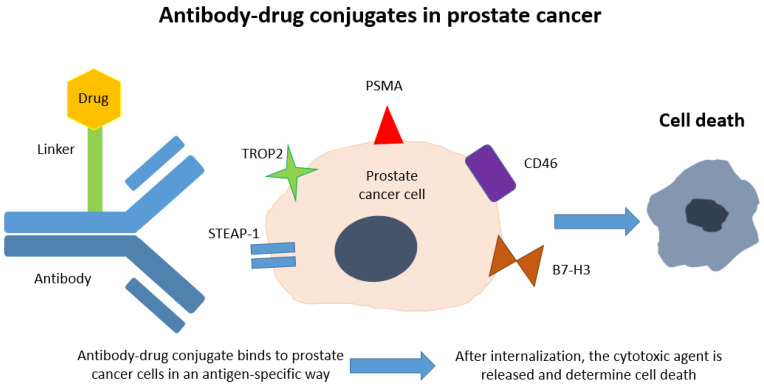
Cartoon representing the mechanism of action of ADCs in prostate cancer.

Although ADCs have already achieved practice-changing clinical outcomes in some malignancies, as trastuzumab deruxtecan for breast cancer, studies regarding their use in mCRPC treatment are continuously growing nowadays. Regarding mCRPC therapy, researchers are focusing on STEAP1, TROP2, PSMA, CD46 and B7-H3 as optimal antigens, which may be targeted by ADCs. In the sections below we describe the role of these antigens in prostate cancer and the clinical trials ongoing in this setting (Figure 1).

### 4.1. STEAP1

STEAP1 (six-transmembrane epithelial antigen of the prostate 1), a transmembrane channel used as ions/proteins transporter, represents an ideal target for ADC-based therapies because of its high expression in prostate cancer cells whereas it is very low expressed in normal tissues [44]. The molecular mechanisms of STEAP1 activity have been recently elucidated. Enzymatic assays in human cells showed that STEAP1 promotes iron(III) reduction when in STEAP heterotrimers with the intracellular NADPH-binding domain of STEAP4, another member of STEAP family [45]. Knockdown of STEAP1 gene has been correlated with inhibited cell viability and proliferation and enhanced apoptosis in LNCaP prostate cancer line [46]. Furthermore, targeting STEAP1 through a specific single chain antibody blocked gap junctions and resulted in a reduction of 80–90% of the intercellular communications between prostate cancer cells [47].

Of note, imaging through anti-STEAP1 antibody 89Zr-DFO-MSTP2109A revealed high SUV in bone and soft tissue localization from prostate cancer [48]. Bayesian analysis estimated an 86% of histologically positive lesions being true-positive on imaging, without toxicities [48], opening the way to further investigations of anti-STEAP1 antibodies in the diagnostic setting. 

As for ADCs, DSTP3086S is a humanized IgG1 anti-STEAP1 monoclonal antibody linked to the microtubule disrupting agent monomethyl-auristatin E (MMAE). In the phase I trial led by Danila and his group, DSTP3086S showed an acceptable safety profile as well as a significant antitumor activity among men with high STEAP1 expression mCRPC beyond systemic therapies [49]. Sixty-two patients were given >2 mg/kg of DSTP3086S once every 3 weeks; 18% of them presented a ≥50% decline in Prostate-Specific Antigen (PSA), while 6% achieved a radiographic partial response [49].

At present, a phase I study is in course to explore AMG 509, an anti-STEAP1 antibody, in mCRPC patients (NCT04221542, Table 3). The study will enroll 70 patients who are refractory to a novel antiandrogen therapy and have failed not more than 2 taxane regimens. Further clinical trials are required in order to refine knowledge about STEAP1 for the development of novel ADCs and so on.

### 4.2. TROP2

TROP2 (also known as “trophoblast antigen 2”) is a membrane-bound glycoprotein normally expressed in several tissues, including epidermis, breast, cervix, cornea, lung, liver, pancreas, prostate, trophoblast cells or urothelium. This transmembrane antigen is also overexpressed in many malignancies [50]. Among them, TROP2 is upregulated in invasive prostate cancer and its expression promotes a α5β1 integrin-dependent pro-metastatic signaling pathway in cancer cells [51]. Moreover, TROP2 expression has been correlated with neuroendocrine differentiation of prostate cancer cells [52], which confers resistance to standard therapies and is associated with poor outcome [53,54].

TROP2 is targeted by sacituzumab govitecan (IMMU-132), which is an irinotecan active metabolite (SN-38)-based ADC, in which SN-38 is covalently linked to a monoclonal antibody (hRS7) via a hydrolysable CL2A linker. In April 2020, sacituzumab govitecan has received accelerate approval by US FDA for the treatment of metastatic triple-negative breast cancer [55]. Moreover, according to the above mentioned TROP2 upregulation, the clinical activity of this ADC is under investigation in many ongoing trials to improve therapeutic options against several tumors, such as urothelial cancer [56].

As for prostate cancer, an ongoing phase II trial (NCT03725761, Table 3) is assessing sacituzumab govitecan among men with mCRPC progressing on ARSI [8]. This study will include 55 patients and will be concluded in October 2021.

### 4.3. PSMA

**Figure 2:**
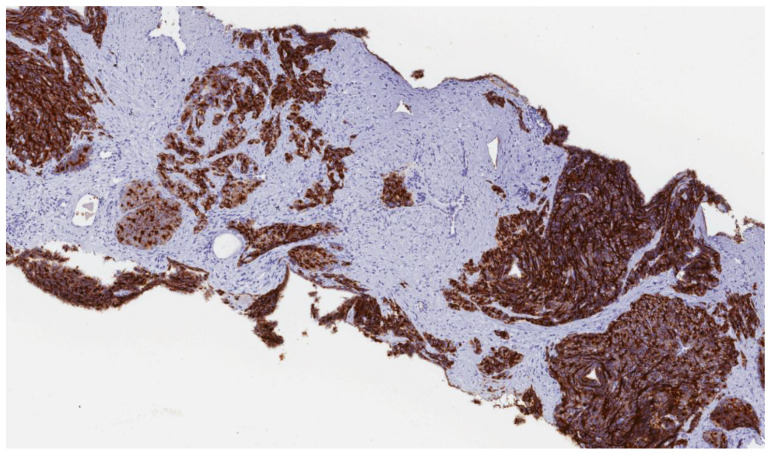
Biopsy sample of prostate tissue infiltrated by prostatic adenocarcinoma, with a strong and diffuse prostate specific membrane antigen (PSMA) expression. (magnification: 4×)

Surely, one of the most well-known and clinically validated markers of prostate cancer is the prostate-specific membrane antigen (PSMA, also known as folate-hydrolase 1), a 100 kD transmembrane glycoprotein with expression properties that allow its use as diagnostic and therapeutic target (Figure 2) [57]. Moreover, PSMA inhibition leads to blockage of phosphoinositide-3 kinase (PI3k) and serine/threonine kinase (AKT) signaling pathways, both of which are meaningful in cancer cells proliferation [58]. First of all, after encouraging results with 131I-marked PSMA ligands, PSMA has been exploited for radioligand-therapy. The so-called lutetium-177 (177Lu-PSMA-617), a beta-emitter radioligand, have not received FDA and EMA approval yet, but it is still central in several studies in order to assess its efficacy and safety [59]. More recently, two PSMA-targeted antibody–drug conjugates have been evaluated in the castration-resistant metastatic setting.

MLN2704 is the result of conjugating the cytotoxic maytansinoid-1 (DM-1) with MLN591, a humanized antibody developed to deliver this payload to PSMA overexpressing cells. Unfortunately, the two MLN2704-focused trials unveiled an unfavorable safety profile with peripheral neuropathy proved as the most disabling toxicity. This adverse event (AE) is a consequence of the instability of ADC, as well as other AEs described [60,61,62]. On the other side, PSMA-ADC is a PSMA-aimed fully humanized monoclonal antibody conjugated to the above-mentioned MMAE, via a di-peptide linker. In 2019, Petrylak et al. published results of a phase I multicenter study: these data shed light on PSMA-ADC clinical activity when it was administered every three weeks at a dose from 1.8 mg/kg to 2.5 mg/kg among mCRPC patients already treated with abiraterone acetate or enzalutamide. Although a lower rate of neurotoxicity has been registered, deconjugation keeps on being a problem in terms of safety, even if a di-peptide linker is used [60,63].

On March 2021, a phase one study will start enrolling mCRPC patients to evaluate ARX517, a PSMA ADC conjugated to microtubule-disrupting toxins AS269 (NCT04662580).

### 4.4. CD46

CD46 is a transmembrane glycoprotein, which acts as a complement regulator by inactivating C3b and C4b [64]. CD46 results crucial for the downregulation of Th1 response by substituting IFNγ + IL-10- CD4+ T cells into IFNγ + IL-10+ cells [64]. Deficiency in CD46 decreases the surface expression of C3b and/or C4b inactivating capacity, leading to uncontrolled complement activation and systemic micro thrombi formation [64].

Basing on the evidence that the expression of CD46 is high in prostate cancer tissue and CRPC but low in normal tissues [65], CD46 represents an ideal target for ADC therapy [66]. CD46 ADC has demonstrated to potently and selectively kill both adenocarcinoma and NEPC cells both in vitro and in vivo [66]. At this regard, a phase I study will enroll 60 participants to assess the safety and efficacy of FOR46, an intravenously administered ADC directed against CD46 in mCRPC patients (NCT03575819, Table 3). The study completion is estimated on 31 December 2021.

### 4.5. B7-H3

B7-H3, also referred to as CD276, is an important immunomodulatory molecule. B7-H3 is a 316-amino-acid-long type I transmembrane protein expressed on the surface of tumor cells, antigen presenting cells, natural killer cells and tumor endothelial cells [67]. In tumor tissues, B7-H3 inhibits immune response as well as promote the migration and invasion, angiogenesis and endothelial-to-mesenchymal transition of tumor cells [67]. It has been shown that B7-H3 expression promotes prostate cancer progression in vivo by reducing myeloid-derived suppressor cell apoptosis [68]. In addition, B7-H3 overexpression correlates with an increased risk of prostate cancer progression [69].

The increasing knowledge on the role of B7-H3 in solid tumors has led to the preclinical development of MGC018, composed by valine-citrulline-seco duocarmycin hydroxybenzamide azaindole conjugated to an anti-B7-H3 humanized IgG1/kappa monoclonal antibody [70]. Currently, a phase I/II trial is investigating MGC018 alone and in combination with anti-PD-1 antibody MGA012 in patients with advanced solid tumors including prostate cancer (NCT03729596).

## 5. Discussion

Treatment of prostate cancer remains a major challenge even if the pathophysiology has become clearer with time. In the last few years, we have witnessed to the enthusiastic results of novel hormone therapies and PARP inhibitors along with the failure of immunocheckpoint inhibitors for the cure of this malignancy. More than 4900 trials are currently ongoing in this setting, reflecting the efforts in finding novel diagnostic and therapeutic approaches for early and advanced stages.

After having reached meaningful outcomes which are progressively changing clinical practice in other malignancies management (i.e., sacituzumab govitecan, trastuzumab deruztecan, enfortumab vedotin, etc.), antibody–drug conjugates (ADCs) are gaining ground as promising treatment approach among patients with metastatic prostate cancer.

ADCs can specifically identify overexpressed antigens on cancer cells, release anticancer drugs and selectively kill tumor cells. However, many antigens are also present on normal cells, thus diverting the targeting molecules to cancer cells. The research for the best candidate for ADC therapy in prostate cancer has led to a series of studies in which STEAP, TROP2, PSMA, CD46 and B7-H3 are the main actors.

The majority of ongoing trials of ADCs in patients with prostate cancer are enrolling subjects with metastatic tumors that have become castration resistant. Nevertheless, exploring ADCs also in the early stages will represent an interesting challenge in future years and will allow assessing the ideal setting for this strategy.

Nowadays, the recent published trials along with the intermediate results of the ongoing studies are highlighting promising efficacy and safety profiles of these agents in the mCRPC therapeutic setting. Keeping in mind the critical parameters in the development of the “ideal” ADC (selection of the targetable antigen, ability to be internalized in the targeted cell after binding to the antigen, drug potency and ADC conjugation stability), physicians have to elect the most suitable antigen in order to develop potentially practice changing compounds.

The growing body of evidences on the sensitivity and specificity of PSMA PET-CT in the management of prostate cancer has led to the rapid worldwide diffusion of this technique, which is becoming the new gold standard. The great attention currently payed to PSMA may represent, in our opinion, a favoring factor for the prevailing of this prostate specific antigen on the other targets for ADC therapy against prostate tumor in future years. Nonetheless, it is interesting to observe the further results assessing the efficacy and safety profile of other prostate cancer specific antigens-based ADCs, such as sacituzumab govitecan along with CD46-based and B7-H3-based ADCs.

Unfortunately, there are still some hurdles that must be overwhelmed to guarantee a revolutionary role for ADCs in the wide therapeutic scenario of prostate cancer. First of all, cancer cells may develop a mechanism of resistance to ADCs which implies the failure or reduction of the treatment, as demonstrated for all the other antitumoral therapies (such as chemotherapy or immunotherapy) [71]. These resistance mechanisms may be present within tumor cells regardless of therapies (primary or de novo resistance) or may be acquired after the beginning of treatments (secondary resistance). Several preclinical trials showed the main causes of ADCs resistance, among which antigen-related phenomena are noteworthy. Structural alterations of the targeted antigens recognized by the antibody component of an ADC may lead to treatment failure, as well as a compensatory decrease in antigens expression levels due to a high exposure to the delivered payload. Of note, transmembrane antigens overexpression may also reduce the exposure of malignant cells to the drug, declining ADCs efficacy. Moreover, ADCs resistance could be connected to faulty lysosomal activity, which has a key role for the cytotoxic payload release, or upregulated drug efflux pumps, or impaired apoptotic pathway. Overcoming the development of ADCs resistance is a critical step for these novel compounds to be clinically successful. Some strategies devised to solve this issue are modifications of the modular structure of ADCs or the linker-cytotoxic component in order to gain brand new compounds to knock cancer cells resistance out [72]. Furthermore, changes in the linker-cytotoxic structure can help ADCs to hit not only antigen-positive cells but also other neighboring cells in the malignant tissue, owing to the “bystander effect” depending on particular linker and drugs combinations that are able to release the payload in the tumor microenvironment. This approach ensures that ADCs kill tumor cells, regardless of the aimed antigen transmembrane expression [72].

Another issue, that limits clinical use of these drugs, is ADCs toxicity. Although these compounds are relatively well tolerated by patients, the expression of the targeted antigen over normal cells surface along with payload toxicity may promote the development of some adverse events, such as gastrointestinal or hematopoietic ones. Several trials designed to investigate PSMA-based ADCs clinical activity have pointed out peripheral neuropathy as the main dose-limiting toxicity. This adverse event appears to be related to the rapid deconjugation of bound payload from the antibody component of ADCs. The rapid deconjugation leads to a premature release of cytotoxic payload in the circulation, decreasing the therapeutic window of ADCs. Nowadays, lots of preclinical studies are ongoing to find novel strategies for the optimization of linker components, trying to overcome this problem [72].

Current advancements in technology are surely progressively contributing to improve target specificity along with the selection of more suitable linkers and payloads, bringing to hopeful new generation ADCs [73]. The conjugate synthesis is profiting from protein engineering and biochemistry improvements. Furthermore, new generation ADCs may incorporate the recently studied “bispecific” monoclonal antibodies, which are designed to aim different targets. Current trials showed a sort of immunomodulatory property of many ADCs, suggesting their administration with immunotherapy in order to potentiate it. All these advances may improve ADCs therapeutic index, bringing to further compounds related to better clinical outcomes.

Looking at the great success reached in other malignancies treatment, we think that ADCs represent a current valid approach to be further explored, which can progressively lead to more durable benefits, in the therapeutic landscape of the prostate cancer too.

In conclusion, although some limitations such as toxicity persist, ADCs represent a current valid approach for prostate cancer to be explored with further studies. The preliminary results obtained by ADCs seem to pave the way to the future inclusion of this approach into the therapeutic armamentarium of this tumor.

## 6. Conclusions

Antibody–drug conjugates represent, still today, an active topic of research as well as a current promising and valid therapeutic strategy for prostate cancer to be explored with further studies. Several trials are still ongoing, investigating the clinical role of novel compounds targeting specific prostate cancer antigens. The available results highlight PSMA-based ADCs as the utmost promising agents, even if there are many obstacles to overc ome. In the next years, technological advancements may provide new-generation ADCs with better efficacy and safety profile, owing to novel and more effective linker agents, antibodies and payloads. Moreover, future ADC-based regimens could be included in prostate cancer treatment courses, testing new drug combinations to optimize therapeutic effects in these patients.

## Figures and Tables

**Table 1:** Recently presented or published phase III clinical trials assessing novel ARSIs in nonmetastatic castration-resistant setting.

Study [Author, Year]	Treatment	Clinical Setting	MFS Benefit	OS Benefit	Adverse Events of Any Grade
**PROSPER** [Hussain et al., 2018] [12,13]	Enzalutamide + ADT (vs. placebo + ADT)	nmCRPC	36.6 months vs. 14.7 months (*p* < 0.001)	67.0 months vs. 56.3 months (*p* = 0.001)	More frequently (Enza vs. placebo): - Fatigue (in 33% vs. 14%) - Hypertension (in 12% vs. 5%) - Major cardiovascular events (in 5% vs. 3%) - Mental impairment disorders (in 5% vs. 2%)
**SPARTAN** [Smith et al., 2018] [15,16]	Apalutamide + ADT (vs. placebo + ADT)	High-risk nmCRPC	40.5 months vs. 16.2 months (*p* < 0.001)	Median OS not reached in the Apa or the placebo group. 25% reduction in the risk of death (HR for Apa vs. placebo, 0.75; 95% CI 0.59–0.96; *p* = 0.0197) [16]	More frequently (Apa vs. placebo): - Fatigue (in 30.4% vs. 21.1%) - Hypertension (in 24.8% vs. 19.8%) - Rash (in 23.8% vs. 5.5%) - Diarrhea (in 20.3% vs. 15.1%)
**ARAMIS** [Fizazi et al., 2019] [17,18]	Darolutamide + ADT (vs. placebo + ADT)	High-risk nmCRPC	40.4 months vs. 18.4 months (*p* < 0.001)	Percentage of pts alive at 3 years: 83% vs. 77% (*p* = 0.003) [18]	More frequently (Daro vs. placebo): - Fatigue (in 13.2% vs. 8.3%) - Hypertension (7.8% vs. 6.5%) - Cardiac arrhythmia (7.3% vs. 4.3%) - Bone fracture (in 5.5% vs. 3.6%)

Abbreviations: ADT: androgen-deprivation therapy; nmCRPC: nonmetastatic castration-resistant prostate cancer; MFS: metastasis-free survival; OS: overall survival; Enza: enzalutamide; Apa: apalutamide; Daro: darolutamide; CI: confidence intervals; HR: hazard ratio; pts: patients; ARSIs: androgen receptor signaling inhibitors.

**Table 2:** Pivotal phase III trials published from 2004 to 2020 that have radically changed the therapeutic scenario of metastatic prostate cancer, both in hormone-sensitive and castration-resistant context.

Study [Author, Year]	Treatment	Clinical Setting	OS Benefit	PFS Benefit	Adverse Events of any Grade
**TAX 327** [Tannock et al., 2004] [22]	Docetaxel * (vs. mitoxantrone)	mCRPC	18.9 months vs. 16.4 months (*p* = 0.009)	-	More frequently (dtx vs. mtx): - Alopecia (65% vs. 13%) - Fatigue (53% vs. 35%) - G3/G4 neutropenia (32% vs. 22%) - Diarrhea (32% vs. 10%) - Sensory neuropathy (30% vs. 7%)
**SWOG 99–16** [Petrylak et al., 2004] [23]	Docetaxel + estramustine (vs. mitoxantrone)	mCRPC	17.5 months vs. 15.6 months (*p* = 0.02)	6.3 months vs. 3.2 months (*p* < 0.001) in terms of TTP	More toxicity due to the addition of estramustine
**CHARTEED** [Sweeney et al., 2015] [24]	Docetaxel + ADT (vs. ADT alone)	High volume mHSPC	57.6 months vs. 44.0 months (*p* < 0.001)	33.0 months vs. 19.8 months (*p* < 0.001) in terms of cPFS	More frequently with dtx + ADT (only high grade described): - Fatigue (4.1%) - Febrile neutropenia (3.8%) - G3/G4 neutropenia (3.1%) - Diarrhea (1%)
**TROPIC** [De Bono et al., 2010] [25]	Cabazitaxel (vs. mitoxantrone)	mCRPC post- docetaxel	15.1 months vs. 12.7 months (*p* < 0.0001)	2.8 months vs. 1.4 months (*p* < 0.0001)	More frequently (caba vs. mtx): - Anemia (97% vs. 81%) - Neutropenia (94% vs. 88%) - Thrombocytopenia (47% vs. 43%) - Diarrhea (47% vs. 11%)
**CARD** [De Wit et al., 2019] [26]	Cabazitaxel (vs. abiraterone acetate + prednisone or enzalutamide)	mCRPC after docetaxel and abiraterone acetate + prednisone or enzalutamide	13.6 months vs. 11.0 months (*p* = 0.008)	8.0 months vs. 3.7 months (*p* < 0.001)	More frequently (caba vs. abi/enza): - Fatigue (53.2% vs. 36.3%) - Diarrhea (39.7% vs. 6.5%) - Infection (33.7% vs. 20.2%) - Musculosketal pain (27.0% vs. 39.5%)
**COU-AA-301** [De Bono et al., 2011] [27]	Abiraterone acetate + prednisone (vs. placebo)	mCRPC post- docetaxel	15.8 months vs. 11.2 months (*p* < 0.001)	5.6 months vs. 3.6 months (*p* < 0.001) in terms of rPFS	More frequently (abi vs. placebo): - Fatigue (47% vs. 44%) - Nausea (33% vs. 33%) - Back pain (33% vs. 36%) - Anemia (25% vs. 28%)
**COU-AA-302** [Ryan et al., 2013] [28,29]	Abiraterone acetate + prednisone (vs. placebo)	mCRPC pre- docetaxel	34.7 months vs. 30.3 months (*p* = 0.003)	16.5 months vs. 8.3 months (*p* < 0.001) in terms of rPFS	More frequently (abi vs. placebo): - Fluid retention (30% vs. 23%) - Hypertension (19% vs. 11%) - Hypokalemia (16% vs. 11%) - Cardiac disorders (16% vs. 14%)
**LATITUDE** [Fizazi et al., 2017] [30]	Abiraterone acetate + prednisone (vs. placebo)	High-risk mHSPC	53.5 months vs. 36.5 months (*p* < 0.0001)	33.0 months vs. 14.8 months (*p* < 0.001) in terms of rPFS	More frequently (abi vs. placebo): - Hypertension (37% vs. 22%) - Hypokalemia (20% vs. 4%) - Increased ALT (16% vs. 13%) - Hyperglycemia (13% vs. 11%)
**AFFIRM** [Scher et al., 2012] [31]	Enzalutamide (vs. placebo)	mCRPC post- docetaxel	18.4 months vs. 13.6 months (*p* < 0.001)	8.3 months vs. 2.9 months (*p* < 0.001) in terms of rPFS	More frequently (enza vs. placebo): - Fatigue (34% vs. 29%) - Diarrhea (21% vs. 18%) - Hot flash (20% vs. 10%) - Musculoskeletal pain (14% vs. 10%)
**PREVAIL** [Beer et al. 2014] [32]	Enzalutamide (vs. placebo)	mCRPC pre- docetaxel	36.0 months vs. 31.0 months (*p* < 0.001)	65% vs. 14% at 12 months (*p* < 0.001) in terms of rPFS	More frequently (enza vs. placebo): - Fatigue (52% vs. 35%) - GI events (49% vs. 42%) - Hypertension (18% vs. 4.2%) - Fall (16% vs. 5.1%)
**ARCHES** [Armstrong et al., 2019] [33]	Enzalutamide + ADT (vs. placebo + ADT)	mHSPC	OS data still immature	NR vs. 19.0 months (*p* < 0.001)	More frequently (enza vs. placebo): - Hot flash (27.1% vs. 22.3%) - Fatigue (19.6% vs. 15.3%) - Arthralgia (12.2% vs. 10.6%) - Hypertension (8% vs. 5.6%)
**ENZAMET** [Davis et al., 2019] [34]	Enzalutamide + ADT (vs. 1st generation NSAA + ADT)	mHSPC	80% vs. 72% at 3 years according to Kaplan–Meier estimates (*p* = 0.002)	68% vs. 41% at 3 years according to Kaplan–Meier estimates (*p* < 0.001) in terms of cPFS	More frequently (enza vs. 1st generation NSAA): - G2 fatigue (25% vs. 14%) - G3/G4 Hypertension (8% vs. 4%) - G3/G4 Neutropenia (6% vs. 3%)
**PROFOUND** [De Bono et al., 2020] [35]	Olaparib (vs. abiraterone acetate + prednisone or enzalutamide)	mCRPC (Cohort A: at least one alteration in BRCA1/2, or ATM; cohort B: alterations in any of 12 other prespecified genes)	- Cohort A: 18.5 months vs. 15.1 months (*p* = 0.02) - Overall population (Cohorts A and B): 17.5 months vs. 14.3 months (*p* = 0.02)	- Cohort A: 7.4 months vs. 3.6 months (*p* < 0.001) - Overall population (Cohorts A and B): 5.8 months vs. 3.5 months (*p* < 0.001)	More frequently (Ola vs. placebo): - Anemia (46% vs. 15%) - Nausea (41% vs. 19%) - Fatigue (41% vs. 32%) - Decreased appetite (30% vs. 18%)

Abbreviations: mHSPC: metastatic hormone-sensitive prostate cancer; mCRPC: metastatic castration-resistant prostate cancer; OS: overall survival; PFS: progression-free survival; cPFS: clinical PFS; rPFS: radiological PFS; TTP: time to progression; ADT: androgen deprivation therapy; dtx: docetaxel; mtx: mitoxantrone; caba: cabazitaxel; abi: abiraterone acetate; enza: enzalutamide; NSAA: –steroidal anti-androgen; ola: olaparib; GI: gastro-intestinal; G: grade; NR: not-reached. *: data presented for the experimental arm in which docetaxel were administered every 3 weeks; TAX-327 trial had an additional arm where patients were treated with weekly docetaxel regimen, not shown in this table.

**Table 3:** The clinical trial data of antibody–drug conjugates in prostate cancer.

Drug	Target	Cured Disease	Strategy	Phase	NCT Number	Estimated Completion Date
AMG 509	STEAP1	Prostate cancer refractory to a novel antiandrogen therapy and not more than 2 taxane regimens	Single agent	1	NCT04221542	October 2025
Sacituzumab govitecan (IMMU-132)	TROP2	mCRPC progressing on ARSI	Single agent	2	NCT03725761	October 2021
ARX517	PSMA	mCRPC	Single agent	1	NCT04662580	August 2024
FOR46	CD46	mCRPC	Single agent	1	NCT03575819	December 2021
MGC018	B7-H3	Advanced solid tumors including prostate cancer	Single agent or with anti-PD-1 antibody MGA012	1/2	NCT03729596	May 2025

Abbreviations: ARSI = Androgen Receptor Signaling Inhibitor; mCRPC = metastatic Castration Resistant Prostate Cancer.
